# Employees and the National Insurance Act

**Published:** 1912-06-29

**Authors:** 


### NATIONAL INSURANCE ACT DEPARTMENT.

### EMPLOYEES AND THE NATIONAL INSURANCE ACT.

### XVII.—Awakened Interest in Health Insurance.

We have very forcible evidence this week of the awakening of interest that has taken place throughout the country, with reference to the National Insurance Act, in the large increase in the number of letters of inquiry that have reached us. So far as space allows we are furnishing answers to these inquiries, and it is gratifying to us that our advice is being sought in this matter. It is, perhaps, little wonder that we should be asked for information seeing that the Act comes into operation in a fortnight's time, and it is admittedly one of the most complicated that has been placed on the Statute Book in this country for at least one hundred years. We learn that the Insurance Commissioners are receiving about 3,000 letters of inquiry daily, and that almost as many verbal inquiries are made at the offices of the Commission. The Commissioners admit that they are themselves unable to furnish definite answers to certain of these inquiries, and we understand that they are proceeding with some test cases in the Courts to decide some of the most difficult problems. The principal difficulties for the moment are in regard to the construction to be placed on the words "employed persons." These are defined in the Act, but conditions of employment differ so much throughout the country that it was hardly to be expected that a somewhat hastily drafted measure would cover all cases. There are further difficulties foreshadowed in connection with the special rates of contribution where low rates of wages are paid to persons over twenty-one years of age. These difficulties apply more particularly to the piece-worker, whose earnings vary from week to week. The question is also being freely discussed as to the exact meaning to be placed on the words "casual labour." There is no doubt that these difficulties, and many others, will be settled before long, and it appears doubtful if they could have been settled in any way except as the result of actual experience.

#### Nurses and Medical Benefit.

In our issue of June 15 we published a letter dealing with the insurance of nurses, in which it was suggested that nurses should defer joining an approved society until arrangements have been made as to their position in the matter of medical benefit. The question raised by our correspondent is an important one, and we had already alluded to it in a previous article, but we venture to think that his conclusion in advocating delay is not warranted. The point is this: that as nurses are already provided with medical attendance and treatment there is no necessity for this particular benefit conferred by the Act so far as they are concerned. By section 13, sub-section 3, the Insurance Committee by which medical benefit is administered may allow persons to make their own arrangements for receiving medical attendance and treatment, in which case the Committee will contribute for such persons sums not exceeding in the aggregate the amounts which the Committee would otherwise have expended in providing medical benefit for them. It will at once be seen that this refers to insured persons generally, whether they are employed contributors as members of approved societies or as deposit contributors or voluntary contributors. The arrangement has, therefore, nothing to do with the particular approved society to which the insured persons may belong, but is a personal matter between them and the local Insurance Committee. These Committees have met for the first time this week, and so far as medical benefit is concerned final arrangements need not be made by them until January next, when the medical benefit first comes into operation. The decision as between becoming a member of an approved society and a deposit contributor must be made in any case before October next in the case of employed contributors, and, further, we feel convinced that joint action by members of such a society as the Nurses' Insurance Society is much more likely to carry weight with the Insurance Committees than individual action on the part of nurses. We, therefore, advise all female hospital nurses not to delay their applications for membership of that society.

#### A Letter from the Insurance Commissioners.

We have received a letter from the Insurance Commissioners, in which it is pointed out that the use of the terms "Post Office Contributors" or "Post Office Depositors" have frequently been used by public speakers and in the press when reference was intended to "Deposit Contributors"—that is, persons who do not join an approved society. This has given rise to misunderstanding, especially on the part of depositors in the Post Office Savings Bank. The Postmaster-General and the Insurance Commissioners wish it to be generally known that the National Insurance Act does not affect the Post Office Savings Bank system in any way, and that "Post Office Savings Bank Depositors" and "Deposit Contributors" have no connection at all with each other.

We do not recollect having used either of the terms mentioned as having led to misunderstanding, but we would emphasise the remarks of the Insurance Commissioners lest any of our readers should have misconstrued any of our remarks.

#### Exception from Insurance.

A correspondent has called our attention to a very important matter affecting the interests of a large number of nurses.

Under the First Schedule, Part II. (*b*), it is possible to be excepted from insurance if the "employment is under the Crown or any local or other public authority where the Insurance Commissioners certify that the terms of the employment are such as to secure provision in respect of sickness and disablement on the whole not less favourable than the corresponding benefits conferred by the Act."

A circular has been sent by the Insurance Commissioners to all these public authorities, in which, amongst other questions, they ask them to ascertain the views of the various classes of persons in their employ, including, of course, nurses.

The matter is one of considerable urgency, and it is clear that the nurses, who will be affected by the decision of the local or other public authority in whose service they are employed, should consider the matter most carefully, not only from the point of view of the present payments, but also as regards the ultimate benefits.

It is to be noted that the exception only refers to "employment under the Crown or any local or other public authority," so that it would seem that voluntary hospitals do not come within the scope of the provision. It must also be pointed out that the Insurance Commissioners must be satisfied that "the terms of employment are such as to secure provision in respect of *sickness and disablement* on the whole not less favourable than the corresponding benefits under the Act." This is satisfactory so far as it goes, but no mention is made of medical, maternity, and sanatorium benefits, and by acceding to such exceptions the nurses will lose the right to these benefits so long as they remain in the service of the institution which is excepted.

Our correspondent raises the further point as to the position of the nurse on leaving the service of the particular institution. This is, perhaps, the principal difficulty. If on leaving the service of the accepted authority a nurse becomes employed, within the meaning of the Act, she will have to be insured, and if the change of situation does not take place within twelve months of the commencement of the Act, she will either have to submit to reduced benefits or pay a higher contribution. These reductions of benefit or higher contributions will depend upon the age on entry into insurance, and we gather that at certain ages they will be quite large sums.

It, therefore, appears to us that the best interests of the nurses will be served by declining to agree to any such proposal for exception, and that they should instead, where full provision is already made for adequate sickness benefit, apply to their approved society for a variation of the benefits in accordance with section 13—a matter with which we have already dealt in the course of our articles.

### Answers to Correspondents.

### VOLUNTARY CONTRIBUTORS SHOULD ENTER INSURANCE BEFORE JANUARY 15, 1913.

G. M.—It would appear from the information you send us that you do not come within the description of an employed person within the meaning of the Act. If this be so you would not be compelled to be insured, but you could become a voluntary contributor. As such your contribution would have to be paid by yourself in full, and as your present age is above forty-five the rate of contribution will be more than 6d. a week. At age of fifty last birthday the weekly contribution for female voluntary contributors is 9½d., if you join before January 15 next. If you do not join until after that date your contribution will be higher, not only because of the advance in age, but also because specially favourable terms are offered under the Act to those who enter as voluntary contributors within six months of its coming into operation. As you mention that you are insured in the Prudential it would probably be your best course to communicate with the agent who calls on you or with the company direct. You are doubtless aware that that company has formed a separate approved society for women for the administration of the National Insurance Act.

### THE DISADVANTAGE OF BECOMING A DEPOSIT CONTRIBUTOR.

Miss M. E., Aintree.—If you become a deposit contributor you will not enjoy the benefit of insurance. You will only be able to draw on the funds paid by yourself and on your behalf, supplemented by the State grant, for so long as there is money in hand. If, on the other hand, you join an approved society and become entitled to the benefits through sickness, no matter how soon after you become so entitled, you will be able to draw the benefits so long as you are incapable of work. Another most important point is this, that in order to give full benefits to persons above the age of sixteen at the commencement of the Act a special allowance is made to an approved society, called a reserve value, which at some ages is worth more than £10. This is not provided in the case of a deposit contributor. Benefits payable on death are excluded from the Act, and cannot be received through an approved society, but as a small concession to the deposit contributor it is provided that, in the case of women, one-half of the sum standing to her credit in the Post Office Fund shall be paid on her death to the person she may have nominated, or, failing such nomination, to her next-of-kin.

### APPROVED BENEFIT SOCIETY FOR NURSES.

M. B.—Write for full particulars to the Secretary, Nurses' Insurance Society, 15 Buckingham Street, Strand, London, W.C. It is important that all the members of your staff should join an "approved society" before the date (July 15) when the Insurance Act comes into force. The above is the only approved society open to none but women nurses.

Miss M. S., Folkestone.—Write to the Secretary, Nurses' Insurance Society, 15 Buckingham Street, Strand, London, W.C., for a proposal form to joint this society, which is open to none but women nurses. It is important to become a member before the Act comes into force on July 15.

Puzzled.—Is a charwoman who is engaged to come regularly on one or two days a week for a good part of the year, but not the whole year, and whose wages are daily and paid each time she comes, "casual labour," and so exempt or not?—It was held in a case decided in 1908 that employment such as that mentioned is *not* casual labour. To come within the description casual labour it is apparently necessary for the employment to be irregular and by a separate hiring each time.

**The Questions of "A. B.," "Puzzled," and "Passive Resister," are answered on page 338.**

NOTE:—Questions and Correspondence on all doubtful points are invited, and should be addressed to the Editor, The Hospital, 28 Southampton Street, Strand, W.C., and marked "Insurance."

